# UVB Exposure of Farm Animals: Study on a Food-Based Strategy to Bridge the Gap between Current Vitamin D Intakes and Dietary Targets

**DOI:** 10.1371/journal.pone.0069418

**Published:** 2013-07-24

**Authors:** Alexandra Schutkowski, Julia Krämer, Holger Kluge, Frank Hirche, Andreas Krombholz, Torsten Theumer, Gabriele I. Stangl

**Affiliations:** 1 Institute of Agricultural and Nutritional Sciences, Martin-Luther-University Halle-Wittenberg, Halle (Saale), Germany; 2 Fraunhofer Institute for Mechanics of Materials IWM, Halle (Saale), Germany; University of Houston, United States of America

## Abstract

Vitamin D deficiency is a global health problem. This study aimed to investigate the efficacy of ultraviolet (UV) B radiation for improving vitamin D_3_ content of eggs and meat. In a two-factorial design hens that received diets with 0 (-D_3_) or 3,000 IU (+D_3_) vitamin D_3_/kg were non-exposed (-UVB) or exposed to UVB radiation (+UVB) for 3 h daily over 4 weeks. Data show that UVB radiation was very effective in raising the vitamin D_3_ content of egg yolk and meat. Egg yolk from +UVB/−D_3_ hens had a higher vitamin D_3_ content (17.5±7.2 µg/100 g dry matter (DM)) than those from the –UVB/+D_3_ group (5.2±2.4 µg/100 g DM, *p*<0.01). Vitamin D_3_ content in egg yolk of vitamin D_3_-supplemented hens could be further increased by UVB radiation (32.4±10.9 µg/100 g DM). The content of 25-hydroxyvitamin D_3_ (25(OH)D_3_) in the egg yolk also increased in response to UVB, although less pronounced than vitamin D_3_. Meat revealed about 4-fold higher vitamin D_3_ contents in response to UVB than to dietary vitamin D_3_ (*p*<0.001). In conclusion, exposure of hens to UVB is an efficient approach to provide consumers with vitamin D_3_-enriched foods from animal sources.

## Introduction

Vitamin D_3_ deficiency is a global health problem that has considerable impact on health [1], [2], [3]. It is suggested that up to 50% of young adults suffer from vitamin D insufficiency worldwide [4]. Vitamin D_3_ promotes calcium and phosphate absorption in the intestine, decreases the clearance of these minerals from the kidney and is needed for bone mineralization and bone growth [5], [6]. In the last years, more attention has been paid to vitamin D_3_ due to its multiple health benefits. More than 220 genes are identified that significantly changed in expression in response to vitamin D_3_ [3], particularly those that are involved in cell proliferation, cell differentiation, and immune function [5], [7], [8], and vitamin D deficiency is associated with several diseases such as cancer and autoimmune disorders [3], [7], [9]. Thus, the maintenance of an adequate vitamin D_3_ status seems to provide a great preventive health potential. The main source (80–90%) of vitamin D_3_ is the endogenous synthesis of vitamin D_3_ in the skin by exposure to natural sunlight, whereas nutrition contributes to only 10–20% of the vitamin D_3_ supply [6], [10]. Failing outdoor activities, seasonal variations, air pollution, pigmented skin, and the use of sunscreens affect the efficacy of UVB radiation for cutaneous vitamin D_3_ synthesis. Therefore, an increasing number of people depend on dietary sources of vitamin D_3_ to prevent vitamin D_3_ deficiency or inadequacy. With the exception of fatty fish species, such as salmon and mackerel, and fish liver oils [11], most natural foods contain very low amounts of vitamin D_3_ and are not capable of improving vitamin D_3_ status or fulfilling the recommendations for diet intake of vitamin D_3_. Based on a report of the U.S. Institute of Medicine, vitamin D_3_ is recommended in daily amounts of 15 µg for people younger than 71 years in the USA and Canada [12]. Recommendations for vitamin D_3_ intake in different European countries range between 5 and 20 µg daily for adult men and women [13]. In most of the European countries, the recommended amounts of vitamin D_3_ were not met by the intake of natural foods [14]. Therefore, food-based strategies need to be developed to improve vitamin D_3_ status. In the United States and in Canada, a series of industrial produced foods were fortified with vitamin D [15]. In Europe, vitamin D fortification of food is highly regulated and critically discussed. A novel approach to enrich foods with specific nutrients is the “bio-addition”; thereby, foods are fortified through the addition of nutrients to animal feed during livestock farming production, or manipulation of post-harvest food processes. Eggs are widely and regularly consumed, and offer an interesting target for vitamin D_3_ fortification. However, with respect to vitamin D, it is not allowed to fortify animal feed with vitamin D beyond a defined maximum. We therefore came up with the idea that UVB exposure of farm animals such as laying hens might become a promising option to further improve the vitamin D content of foods from animal origin. In an initial experiment we could show that chickens whose upper part of their body was exposed to UVB did not produce vitamin D-enriched eggs [16]. Current analysis from our research group showed that most of the 7-dehydrocholesterol (7-DHC), the pre-cursor and limiting factor for vitamin D_3_ synthesis, was located in the unfeathered skin of the chicken legs. Based on this finding, we hypothesized that an UVB exposure which ensured irradiation of the whole chicken body, including legs, should increase the vitamin D content of eggs and meat. In order to assess the effectiveness of a whole body irradiation of chickens in producing vitamin D-enriched eggs and meat, we analyzed the vitamin D content of eggs and meat in response to UVB treatment of chickens that were fed either a vitamin D_3_-deficient diet or a diet that contained the maximum permissible amount of dietary vitamin D. Besides vitamin D_3_ metabolites in plasma, eggs, and meat, the laying performance, and also egg shell quality and bone stability were analyzed. We further investigated the folate status of the animals to rule out pronounced side effects of the UVB treatment, since solar radiation is supposed to affect the folic acid levels [17], [18], [19].

## Materials and Methods

### Comparative Analysis of 7-DHC Concentrations in Different Skin Areas of Chickens

To obtain information about the amounts and distribution of cutaneous 7-DHC in chickens, we analyzed the 7-DHC concentrations in skin of comb, wattles, unfeathered and feathered legs, and wing of 8 vitamin D_3_-adequately supplied Lohmann layers with an age of 21 weeks. Prior to skin sample preparation feathers were plucked and the skin was dissected free from underlying muscle and fat. Skin samples (approximately 2 × 2 cm) were then snap frozen in liquid nitrogen and stored at −80°C until 7-DHC analysis. Sample treatment and analysis of the 7-DHC concentration in skin is described below.

### Animals and Treatment

The experiment was conducted with 36 Lohmann layers with an initial age of 27 weeks and an average body weight of 1777 g (±141 g). Before starting the experiment, hens were fed a standard diet containing 2,500 IU vitamin D_3_/kg for 2 weeks. Then, the hens were randomly assigned into four groups of 9 hens each. The hens were individually housed in an environmentally controlled room at 16°C and light (30 lx) from 6∶00 a.m. to 8∶00 p.m. All hens were fed a diet that consisted of (g/kg diet) wheat (470), extracted soy bean meal (220), corn (100), barley (68.2), calcium carbonate (85), soybean oil (30), dicalcium phosphate (13), vitamin and mineral mix (10), sodium chloride (2) and DL methionine (1.8). Except vitamin D_3_, vitamins and minerals were added according to the recommendations of the GfE [20]. From the 36 layers, 18 hens received a diet without any vitamin D_3_ (0 IU vitamin D_3_/kg; vitamin D_3_-deficient diet, -D_3_), the other 18 hens were fed a diet supplemented with 3,000 IU vitamin D_3_ (Molekula, Gillingham, U.K.) per kg diet (vitamin D_3_-adequate diet, +D_3_). The diets were calculated on the basis of GfE recommendations for laying hens and contained 11.6 MJ/kg [20]. All diets were fed over a period of 4 weeks. Feed and water from nipple drinkers were available ad libitum during the whole experiment. The experimental procedure was performed according to the established guidelines for care and handling of laboratory animals and was approved by the council of Saxony-Anhalt, Germany (No. 42502-3-656 MLU). The hens were weighted once a week. Food intake, laying performance, egg weight and shell quality were monitored weekly.

### UVB Treatment

Two groups of hens (-D_3_/+UVB and +D_3_/+UVB) were exposed to UVB for 3 h daily (from 8∶00 to 8∶30, from 11∶00 to 12∶00, from 14∶00 to 15∶00 and from 16∶30 to 17∶00). The 10 cm long, 23 W UVB lamps (Hobby UV Kompakt Desert 8% UVB, Dohse Aquaristik KG, Gelsdorf, Germany) with equipped heat protection (Dohse Aquaristik KG) were placed near the cage doors to ensure optimal UVB exposure of the hens’ legs. The lamps emitted UVB in ranges of 280 to 310 nm. The UVB radiation dosage at a distance of 20 cm was 76 µW/cm^2^ (according to the manufacturer’s specification). This UVB irradiation intensity corresponds to that of natural sunlight during summer in the Middle Europe (50° latitude) [21]. An UVB opaque board was placed between the UVB-treated and the non-exposed groups to block incidental irradiation. The UVB lamps had no influence on the temperature inside the cages.

### Sample Collection

Blood samples for analysis of vitamin D metabolites, minerals, and folate from each hen were taken at the beginning and at the end of the experiment. The blood was collected in heparinized tubes (Sarstedt, Nümbrecht, Germany) and centrifuged at 1,100 g for 10 min at 4^o^C. Plasma samples were stored at −80°C pending analysis. To determine egg weight and shell quality, eggs from each hen were collected at the beginning and after week 1, week 2, week 3, and week 4 of the experiment. Egg yolk for analysis of vitamin D_3_ and 25(OH)D_3_ was collected from eggs of each hen at the beginning and at the end of the experiment. At the end of the experimental period, the hens were killed by decapitation. Fibularis longus muscle of each hen was removed for quantification of vitamin D_3_ and 25(OH)D_3_, tibiotarsus was excised to measure bone stability, and liver was removed for analysis of folate. All samples were stored at −80°C pending further analysis.

### Analysis of 25(OH)D_3_ and 1,25-dihydroxyvitamin D_3_ (1,25(OH)_2_D_3_) in Plasma

The plasma concentration of 25(OH)D_3_ was determined by coupled liquid chromatography-mass spectrometry (LC-MS/MS) according to Higashi et al. [22]. In brief, plasma samples were mixed with deuterated 25(OH)D_3_, which was solved in acetonitrile, (Chemaphor Incorporation, Ottawa, Canada) as an internal standard and extracted with n-hexane. To the dried residue, 4-phenyl-1,2,4-triazolin-3,5-dione (solved in acetonitrile) was added for derivatization. Subsequently to the addition of ethanol and salvation in the mobile phase, the samples were analyzed by HPLC (Agilent 1100, Agilent Technologies, Waldbronn, Germany) equipped with Hypersil ODS-column 100 × 2 mm^2^, 5 µm (Agilent Technologies), coupled to a MS system (API 2000, Applied Biosystems, Darmstadt, Germany). The detection limit for 25(OH)D_3_ was 3.7 nmol/l. Between run precision data were calculated from 2 control sera. The coefficient of variation for 25(OH)D_3_ was 3.0%.

The plasma concentration of 1,25(OH)_2_D_3_ was determined using a commercially available ELISA kit (IDS, Boldon, U.K.) according to the manufacturer’s protocol.

### Analysis of Calcium and Inorganic Phosphate in Plasma

Calcium in plasma samples was quantified by a colorimetric assay. The test system was based on the formation of a calcium-o-kresolphtalein complex (Analyticon Biotechnologies AG, Lichtenfels, Germany). Prior to analysis, plasma was diluted 1∶4 with 0.9% NaCl to avoid interferences with triglycerides in plasma.

The plasma concentration of inorganic phosphate was measured spectrophotometrically according to the manufacturer’s protocol (Analyticon Biotechnologies AG). The test system was based on the measurement of ammonium molybdate which forms a complex with inorganic phosphate.

### Analysis of 7-DHC in Skin, and Vitamin D_3_, 25(OH)D_3_ in Egg Yolk and Meat

Vitamin D_3_, 25(OH)D_3_, and 7-DHC were determined by LC-MS/MS. In brief, samples were homogenized, deuterated internal standard (D_3_-d_3_, 25(OH)D_3_-d_6_, and ergosterol, Sigma-Aldrich Chemie GmbH, Taufkirchen, Germany) was added and hydrolyzed under alkaline conditions and oxygen exclusion. Samples were extracted with n-hexane and hexane phase was washed with ultrapure water. The vitamin D metabolites were fractionated using HPLC (Agilent 1100 HPLC, Agilent Technologies) according to Mattila et al. [23]. Further analytical steps were in accordance to those described for 25(OH)D_3_ in plasma. The detection limit was 0.17 µg/100 g for vitamin D_3_, and 0.1 µg/100 g for 25(OH)D_3_. The coefficient of variation for vitamin D_3_ was 3.9%, and 4.6% for 25(OH)D_3_.

### Analysis of Egg Shell Thickness and Stability

Thickness of each egg shell was measured by use of a micrometer screw capable of 0.01 mm accuracy. Thickness of three fragments from the equatorial region of each egg shell was averaged. Prior to analysis, shell membranes were removed. The stability of egg shells were determined by an electronically controlled breaking strength tester (Messtechnik Gutsch, Nauendorf, Germany). Values were expressed in Newton (N).

### Analysis of Tibiotarsus Stability

Three-point bending tests were performed to determine the fracture loads. The specimens were tested using a Zwick Z050 electro-mechanical testing machine (Zwick GmbH & Co KG, Ulm, Germany). The loading rate was set to 80 mm/min, the span (distance between the supports) to 80 mm and the radius of the cylindrical supports and the cylindrical loading blocks to 5 mm. The specimens were carried out at 23±2°C and a relative humidity of 50%.

### Analysis of Folate in Plasma and Liver

Folate in plasma and liver samples was quantified using a microbiological test kit containing Lactobacillus rhamnosus coated microtiter plates according to the manufacturer’s protocol (R-Biopharm AG, Darmstadt, Germany). Prior to analysis, liver samples were homogenized and enzymatically hydrolyzed using pancreatin (R-Biopharm AG, Darmstadt Germany).

### Statistical Analysis

Values are expressed as mean ± SD. Statistical analyses were performed using SPSS 20 (IBM, Armonk, NY, USA). Two-way ANOVA was used to compare the effects of UVB irradiation (-UVB vs. +UVB), dietary vitamin D_3_ (vitamin D_3_-deficient diet vs. vitamin D_3_-adequate diet), and their interaction. When two-way ANOVA revealed a significant interaction between UVB and vitamin D_3_, a post-hoc comparison was performed. In case of variance homogeneity, means of the four groups were compared by Tukey’s test, or in case of variance heterogeneity by Games-Howell test. Significances of differences between basal and final means were tested by the paired *t*-test. Means were considered significantly different at *p*<0.05. Values under the detection limit are represented by randomly assigned values.

## Results

### Concentration of 7-DHC Varies Strongly in the Different Skin Areas of Hens

**Figure 1:**
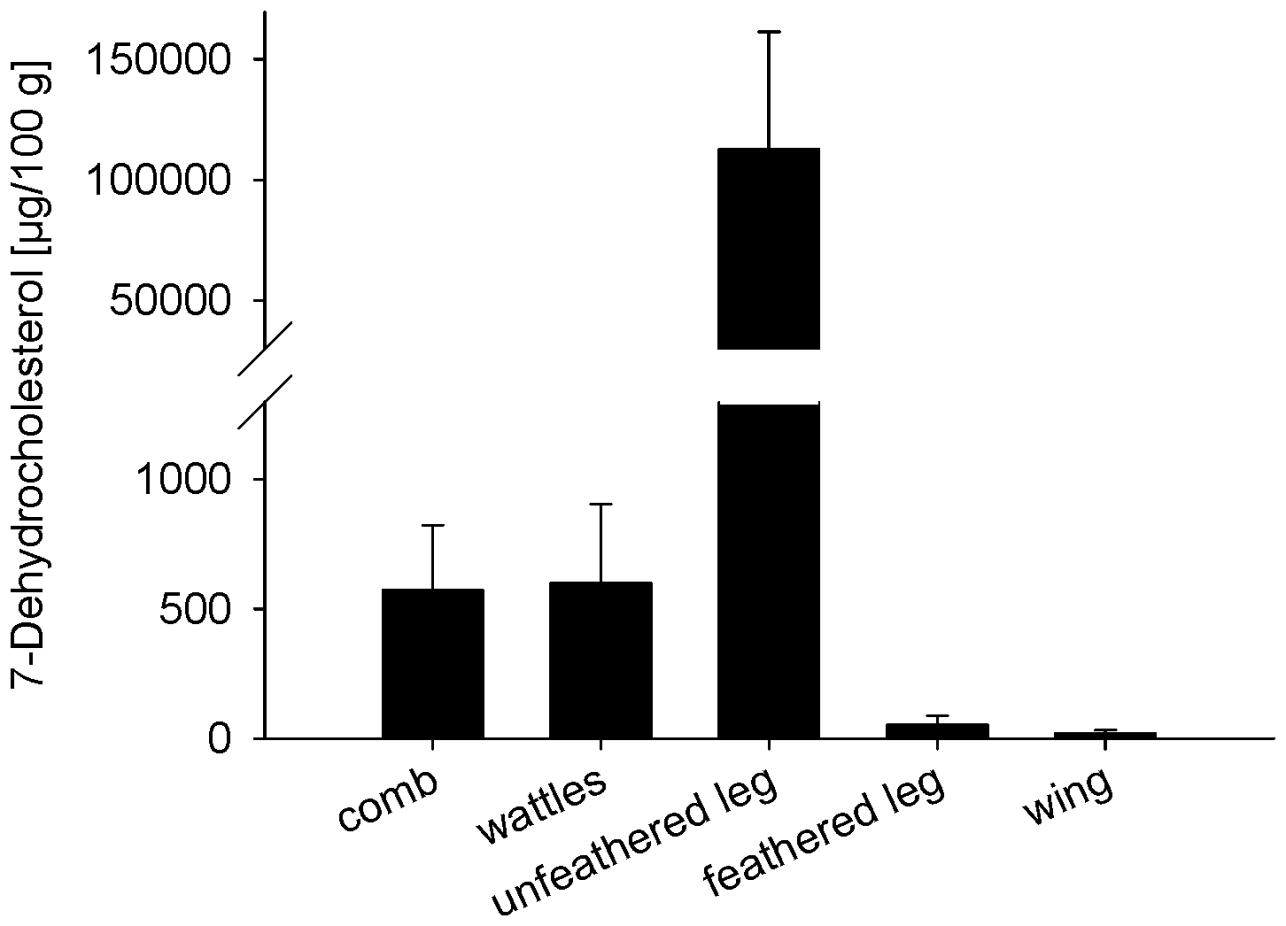
Cutaneous contents of 7-DHC to assess the capacity of different areas of chicken skin to produce vitamin D_3_. Data represent mean ± SD of 7-DHC contents of comb, wattles, unfeathered legs, feathered legs, and wing (n = 8).

7-DHC is the limiting factor for vitamin D_3_ synthesis. To figure out which part of the chicken skin contains most of the vitamin D_3_ precursor molecule and should be inevitably exposed to UVB irradiation to increase vitamin D synthesis, we determined the concentrations of 7-DHC in 5 different skin samples of 8 laying hens by LC-MS/MS. Figure 1 shows large differences in 7-DHC contents between the chosen skin parts. The cutaneous area of the unfeathered legs contained the highest 7-DHC levels, which were on average 190-fold higher than that of the comb. The lowest 7-DHC concentrations were observed in the feathered parts of the skin such as wings and feathered legs. These findings prompted us to mount the UVB lamps in the experimental housing system in a lateral position to ensure an adequate UVB irradiation of skin in the leg area.

### UVB Radiation and Dietary Vitamin D_3_ did not Influence Food Intake and Body Weight

None of the hens showed behavioral peculiarities or symptoms of erythema in response to UVB radiation. Two-way ANOVA did not reveal main and interactive effects of UVB exposure and dietary vitamin D_3_ on daily food intake (-D_3_/−UVB group, 114.1±12.8 g; +D_3_/−UVB group, 118.6±10.0 g; -D_3_/+UVB group, 121.9±7.3 g; +D_3_/+UVB group, 117.3±8.1 g; mean ± SD) and body weight (-D_3_/−UVB group, 1734±151 g; +D_3_/−UVB group, 1797±187 g; -D_3_/+UVB group, 1952±196 g; +D_3_/+UVB group, 1810±97 g; mean ± SD; Table S1).

### Effects of UVB Exposure on Plasma Concentrations of 25(OH)D_3_ and 1,25(OH)_2_D_3_ of hens on a Vitamin D_3_-deficient and Vitamin D_3_-adequate Diet

**Figure 2:**
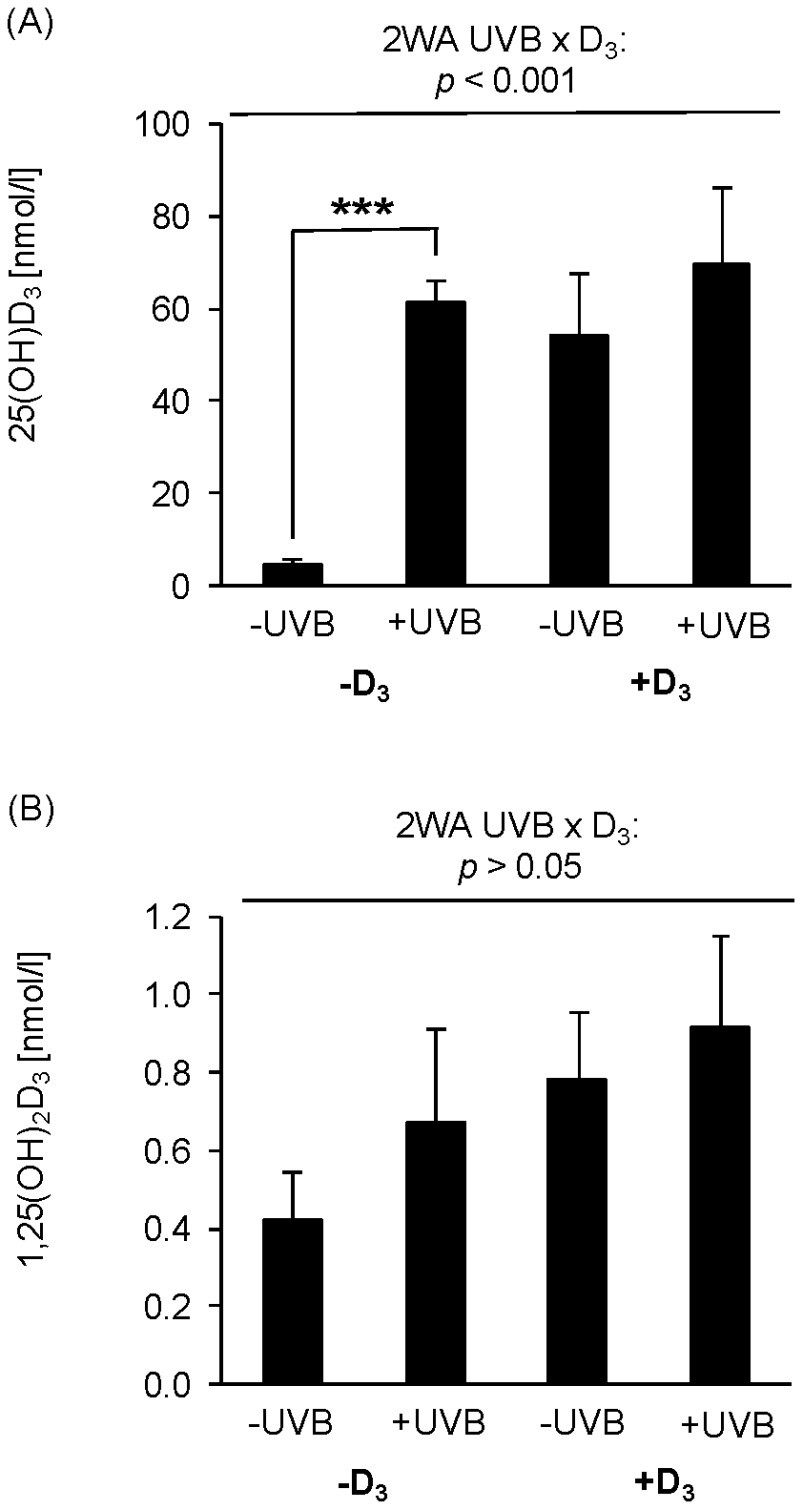
Vitamin D status of chickens in response to UVB exposure and dietary vitamin D_3_. (A) Data in the top panel represent mean ± SD (n = 9) of plasma 25(OH)D_3_ concentrations in non-treated (-UVB) or UVB-treated (+UVB) hens that were fed either a vitamin D_3_-deficient (-D_3_) or vitamin D_3_-adequate diet (+D_3_), respectively. Data were analyzed by two-way ANOVA with the classification factors UVB exposure, vitamin D_3_ in the diet, and the interaction between both factors. Effect of UVB: *p*<0.001, vitamin D_3_: *p*<0.001, UVB × vitamin D_3_: *p*<0.001. Individual means of the groups were compared by post-hoc test. Asterisks within one diet (-D_3_ and +D_3_) indicate a significant difference between -UVB and +UVB groups, ***p<0.001. (B) Data in the bottom panel represent mean ± SD (n = 9) of plasma 1,25(OH)_2_D_3_ concentrations in non-treated (-UVB) or UVB-treated (+UVB) hens that were fed either a vitamin D_3_-deficient (-D_3_) or vitamin D_3_-adequate diet (+D_3_), respectively. Data were analyzed by two-way ANOVA with the classification factors UVB exposure, vitamin D_3_ in the diet, and the interaction between both factors. Effect of UVB: *p*<0.001, vitamin D_3_: *p*<0.001.

To examine the vitamin D status of hens in response to UVB radiation and dietary vitamin D_3_, the plasma concentrations of 25(OH)D_3_ and 1,25(OH)_2_D_3_ were analyzed. Figure 2A and 2B show that the plasma concentration of 25(OH)D_3_ increased much more in response to UVB radiation and dietary vitamin D_3_ than the plasma level of 1,25(OH)_2_D_3_. Two-way ANOVA data reveal a strong interactive effect of UVB exposure and dietary vitamin D_3_ on the plasma concentration of 25(OH)D_3_ (*p*<0.001, Figure 2A, Table S1), and independent effects of UVB exposure (*p*<0.01) and dietary vitamin D_3_ (*p*<0.001) without treatment factor interaction on the circulating plasma level of 1,25(OH)_2_D_3_ (Figure 2B, Table S1). By comparison of the 25(OH)D_3_ plasma levels in response to the treatment factors it was noticeable that UVB treatment was capable of increasing the 25(OH)D_3_ plasma levels in the group of hens on a vitamin D_3_-deficient diet (*p*<0.001) but not in the hens that received the vitamin D_3_-adequate diet (Figure 2A). Supplementation of dietary vitamin D_3_ markedly increased the plasma level of 25(OH)D_3_ in the group which was non-exposed to UVB radiation but not in the group exposed to UVB radiation (*p*<0.001, Figure 2A). Figure 2B shows that hens on a vitamin D_3_-deficient diet that were non-exposed to UVB had the lowest plasma level of 1,25(OH)_2_D_3_ compared to the other groups. Two-way ANOVA data revealed that both treatment factors contributed to increase the 1,25(OH)_2_D_3_ plasma concentration (*p*<0.01, Table S1).

### Plasma Concentrations of Calcium and Inorganic Phosphate were not Affected by UVB Exposure and Dietary Vitamin D_3_

Despite strong differences in vitamin D status, two-way ANOVA did not reveal any significant effects of UVB exposure or dietary vitamin D_3_ on plasma concentrations of calcium (-UVB/−D_3_ group, 6.79±1.66 nmol/l; -UVB/+D_3_ group, 8.11±1.65 nmol/l; +UVB/−D_3_ group, 7.50±1.96 nmol/l; +UVB/+D_3_ group, 8.22±1.52 nmol/l; mean ± SD) and inorganic phosphate (-UVB/−D_3_ group, 1.73±0.37 nmol/l; -UVB/+D_3_ group, 1.89±0.39 nmol/l; +UVB/−D_3_ group, 1.76±0.20 nmol/l; +UVB/+D_3_ group, 1.98±0.39 nmol/l; mean ± SD) (Table S1).

### Effects of UVB Exposure on Vitamin D_3_ and 25(OH)D_3_ in Egg Yolk of Hens on a Vitamin D_3_-deficient and Vitamin D_3_-adequate Diet

**Figure 3:**
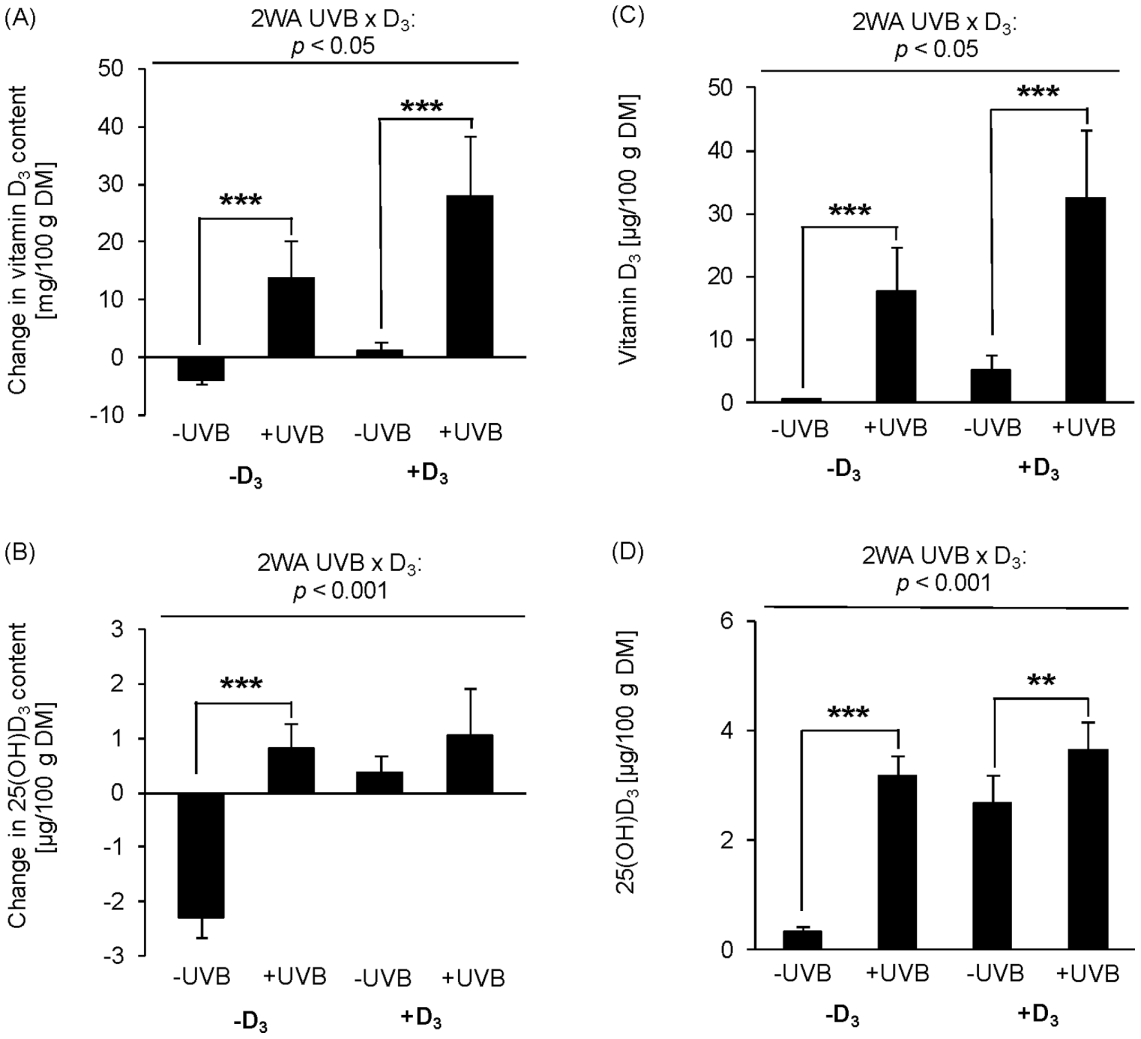
UVB exposure is an effective approach to fortify egg yolk with vitamin D_3_. Changes between basal and final vitamin D_3_ (A) and 25(OH)D_3_ (B) contents in egg yolk dry matter (DM) and final contents of vitamin D_3_ (C) and 25(OH)D_3_ (D) in egg yolk DM and in in non-treated (-UVB) or UVB-treated (+UVB) hens that were fed either a vitamin D_3_-deficient (-D_3_) or vitamin D_3_-adequate diet (+D_3_), respectively. Data represent mean ± SD (n = 9). Data were analyzed by two-way ANOVA with the classification factors were UVB exposure, vitamin D_3_ in the diet, and the interaction between both factors. (A) Effect of UVB: *p*<0.001, vitamin D_3_: *p*<0.001, UVB × vitamin D_3_: *p*<0.05. (B) Effect of UVB: *p*<0.001, vitamin D_3_: *p*<0.001, UVB × vitamin D_3_: *p*<0.001. When two-way ANOVA (2WA) revealed a significant interaction between UVB x vitamin D_3_ individual means of the groups were compared by post-hoc test. Asterisks within one diet group (-D_3_ and +D_3_) indicate a significant difference between -UVB and +UVB groups, *p<0.05, **p<0.01, ***p<0.001.

Figures 3A and 3C show the changes (final - basal) and final contents of vitamin D_3_ in egg yolk in response to UVB exposure of hens on a vitamin D_3_-deficient and vitamin D_3_-adequate diet. Two-way ANOVA revealed significant effects of UVB radiation (*p*<0.001), dietary vitamin D_3_ (*p*<0.001) and an interaction between these two factors (*p*<0.05) on the vitamin D_3_ content of the egg yolk (Table S1). The findings demonstrate that both treatment factors were capable of increasing the vitamin D_3_ content in eggs, even though the UVB irradiation was more effective than the dietary vitamin D_3_ supplementation. Importantly, we found that dietary vitamin D_3_ could increase the vitamin D_3_ content of egg yolk stronger in the group exposed to UVB than in the group non-exposed to UVB radiation. Thus, by far the highest content of vitamin D_3_ in eggs could be obtained with a combination of UVB exposure and dietary vitamin D_3_. Figures 3B and 3D show the changes (final - basal) and final 25(OH)D_3_ contents in egg yolk in response to the dietary vitamin D_3_ and UVB treatment. An interactive effect of dietary vitamin D_3_ and UVB exposure on 25(OH)D_3_ changes (*p*<0.001) and the final 25(OH)D_3_ (*p*<0.001) content of egg yolk was confirmed by two-way ANOVA (Table S1). Main effects for UVB exposure (*p*<0.001) and dietary vitamin D_3_ (*p*<0.001) were also significant (Table S1). As expected, the 25(OH)D_3_ content of eggs decreased compared to baseline if hens on a vitamin D_3_-deficient diet were non-exposed to UVB radiation (Figure 3B, *p*<0.001). UVB irradiation and dietary vitamin D_3_ improved the 25(OH)D_3_ content in egg yolk (two-way ANOVA, *p*<0.001, Figure 3D, Table S1), although the UVB irradiation was marginally more effective than the dietary vitamin D_3_ in increasing the 25(OH)D_3_ content in egg yolk. Interestingly, dietary vitamin D_3_ particularly increased the 25(OH)D_3_ contents of egg yolk in UVB-non-exposed hens on the vitamin D_3_-deficient diet, and to a minor extent in UVB-exposed animals (Figure 3D). Nevertheless, as shown for the vitamin D_3_ content of eggs, the highest 25(OH)D_3_ contents in eggs resulted from a combination of UVB radiation and dietary vitamin D_3_. In all treatment groups, egg white did not show any detectable contents of vitamin D_2_ (detection limit 0.17 µg/100 g) and 25(OH)D_2_ (0.1 µg/100 g) (data not shown).

### Effects of UVB Exposure on Vitamin D_3_ and 25(OH)D_3_ in Fibularis Longus Muscle of Hens on a Vitamin D_3_-deficient and Vitamin D_3_-adequate Diet

**Figure 4:**
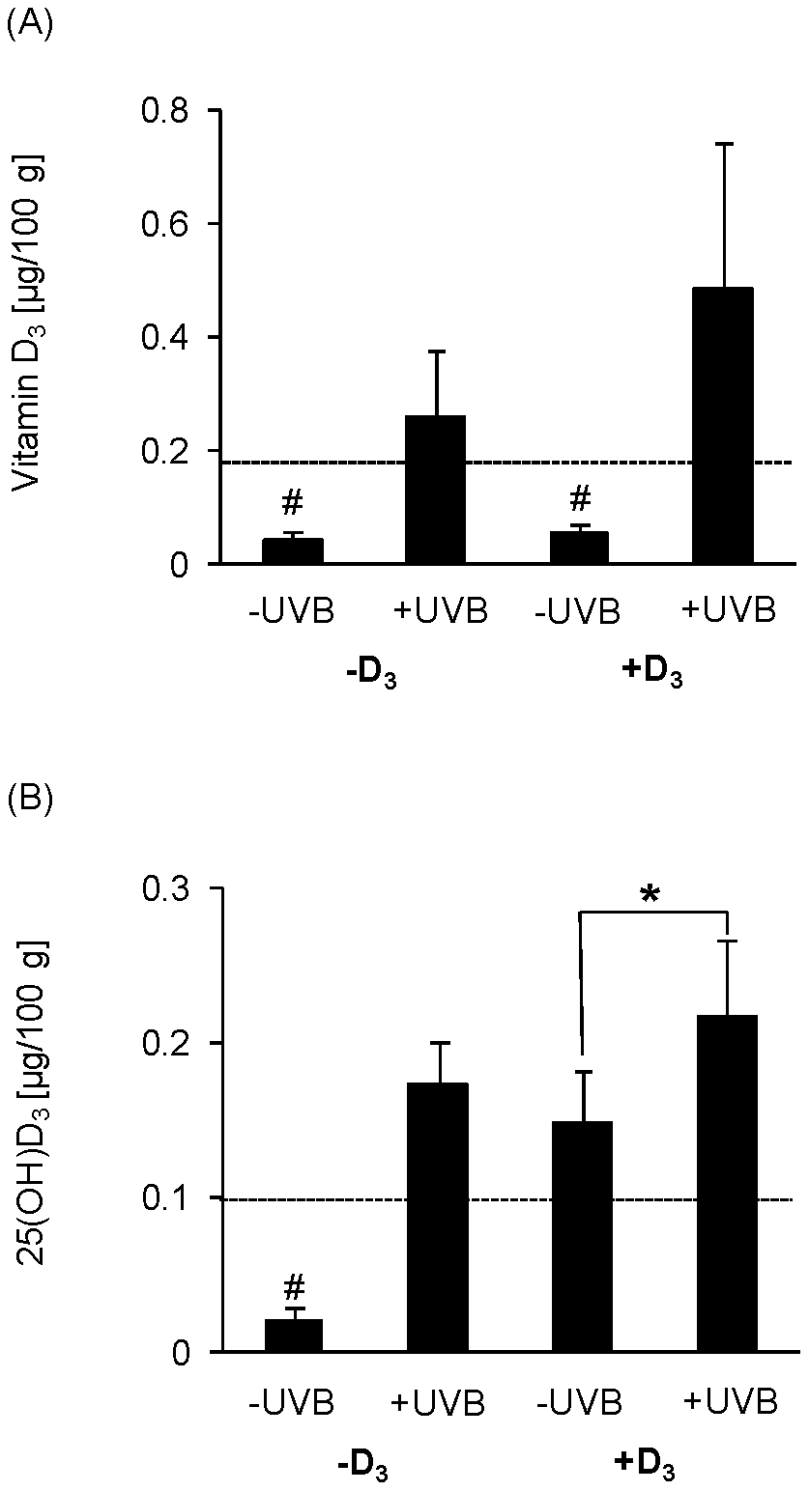
UVB exposure increases vitamin D_3_ content in skeletal muscle. (A) Data in the top panel represent mean ± SD (n = 9) of vitamin D_3_ content in fibularis longus muscle of non-treated (-UVB) or UVB-treated (+UVB) hens that were fed either a vitamin D_3_-deficient (-D_3_) or vitamin D_3_-adequate diet (+D_3_), respectively. Values below the detection limit of 0.17 µg/100g for vitamin D_3_ are represented by randomly assigned values (#). The detection limit is marked by a dotted line (**^…^**). UVB exposure, but not dietary vitamin D_3_ was capable of increasing the vitamin D_3_ content in muscle to values above the detection limit. (B) Data in the bottom panel represent mean ± SD (n = 9) of 25(OH)D_3_ content in fibularis longus muscle of non-treated (-UVB) or UVB-treated (+UVB) hens that were fed either a vitamin D_3_-deficient (-D_3_) or vitamin D_3_-adequate diet (+D_3_), respectively. Values below the detection limit of 0.1 µg/100g for 25(OH)D_3_ are represented by randomly assigned values (#). The detection limit is marked by a dotted line (**^…^**). Individual means of the groups were compared by post-hoc test. Asterisks within one diet group (-D_3_ and +D_3_) indicate a significant difference between -UVB and +UVB groups, *p<0.05.

Irrespective of the vitamin D_3_ in the diet, hens non-exposed to UVB radiation had no detectable vitamin D_3_ contents in their fibularis longus muscles (Figure 4A). UVB irradiation increased the vitamin D_3_ content in the muscles of chickens to values that ranged between 0.16 and 0.96 µg/100 g. By comparison of both UVB-exposed groups, the vitamin D_3_ content of muscle was higher in the group that received the vitamin D_3_-adequate diet than in the group that was fed the vitamin D_3_-deficient diet (*p*<0.05). Figure 4B demonstrates the 25(OH)D_3_ content in the muscles in response to UVB radiation in hens on a vitamin D_3_-deficient and -adequate diet. Similar to the vitamin D_3_ data, hens of the -D_3_/−UVB group had undetectable contents of 25(OH)D_3_ in their muscles. Supplementation with dietary vitamin D_3_ and also UVB exposure slightly increased the muscle contents of 25(OH)D_3_, whereby the UVB-exposed groups reached higher contents in their muscles than the group fed the vitamin D_3_-adequate diet (*p*<0.05).

### Effect of UVB Exposure on Laying Performance, Egg Weight and Egg Shell Quality of Hens on a Vitamin D_3_-deficient and Vitamin D_3_-adequate Diet

**Figure 5:**
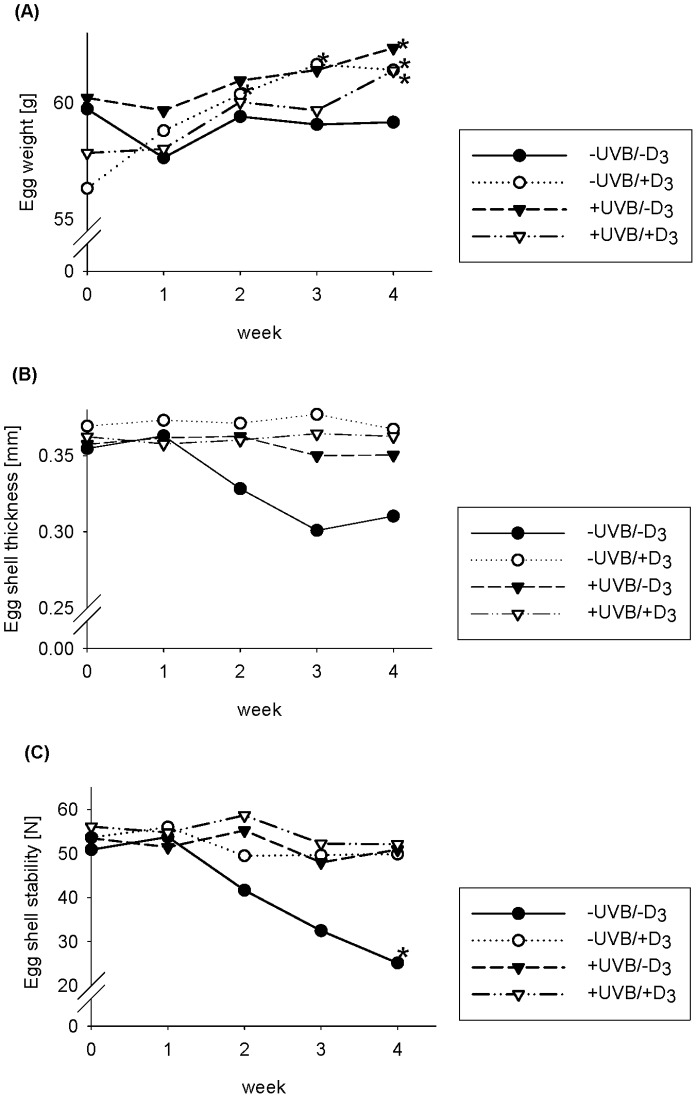
Egg weight and egg shell quality in response to UVB exposure and dietary vitamin D_3_. Effects of UVB exposure (UVB) and dietary vitamin D_3_ (D_3_) on egg weight (A), egg shell thickness (B), and egg shell stability (C) over 4 weeks. Data in the panels represented means (n = 9). Data were analyzed by two-way ANOVA with the classification factors UVB exposure, vitamin D_3_ in the diet, and the interaction between both factors. (A) No significant difference. (B) Effect of vitamin D_3_ (week 2): *p*<0.05, UVB × vitamin D_3_ (week 2): *p*<0.05. Effect of vitamin D_3_ (week 3 and 4): *p*<0.01, UVB × vitamin D_3_ (week 3 and 4): *p*<0.10. (C) Effect of UVB (week 2): *p*<0.05. Effect of UVB (week 3): *p*<0.10, vitamin D_3_ (week 3): *p*<0.10. Effect of UVB (week 4): *p*<0.01, vitamin D_3_ (week 4): *p*<0.05, UVB × vitamin D_3_ (week 4): *p*<0.05. Data were additionally analyzed by paired *t*-test, *significantly different from baseline.

Mean egg production rate (number of eggs per hen and week) was not significantly influenced by the treatment factors, although hens from the -UVB/−D_3_ group showed a slight drop in egg production within the last experimental week compared to the other group (-UVB/−D_3_ group, 6.0±1.6 eggs/week; -UVB/+D_3_ group, 7.0±0.0 eggs/week; +UVB/−D_3_ group, 6.9±0.3 eggs/week; +UVB/+D_3_ group, 7.0±0.0 eggs/week) (Table S1). Two-way ANOVA data show that the mean egg weights at defined times within the 4-week period of the experiment were not significantly influenced by UVB exposure and dietary vitamin D_3_, respectively (Figure 5A, Table S1). Data demonstrate higher egg weights at the end of the 4-week experiment compared to baseline in the groups that were UVB exposed and/or received vitamin D_3_ with their diets (*p*<0.05, paired *t*-test), but not in the -UVB/−D_3_ group (Figure 5A).

Figures 5B and 5C show the egg shell thickness and the egg shell stability in response to UVB radiation and dietary vitamin D_3_ during the 4-week experiment. From the beginning of the second experimental week, egg shell thickness was significantly influenced by dietary vitamin D_3_ (*p*<0.05, two-way ANOVA, Figure 5B), and there was a tendency of an interaction effect on egg shell thickness at the end of the experiment (*p*  = 0.053, two-way ANOVA). Although the -UVB/−D_3_ group showed a trend toward lower egg shell thickness after 3 and 4 weeks of the experiment, paired *t*-test data did not reveal differences compared to baseline. During the experimental period an increasing influence of UVB and dietary vitamin D_3_ on egg shell stability became evident. At the end of the experiment, two-way ANOVA analysis revealed significant main effects of UVB radiation (*p*<0.01) and dietary vitamin D_3_ (*p*<0.05) and a significant interaction between these both factors (*p*<0.05) on egg shell stability (Figure 5C). Compared to baseline, the stability of eggs from the -UVB/−D_3_ group was constantly dropping during the experimental period, and reached a minimum after 4 weeks which was significantly lower compared to baseline (*p*<0.05, paired *t*-test, Figure 5C).

### Effect of UVB Exposure on Tibiotarsus Stability and Folate Status of Hens on a Vitamin D_3_-deficient and Vitamin D_3_-adequate Diet

**Figure 6:**
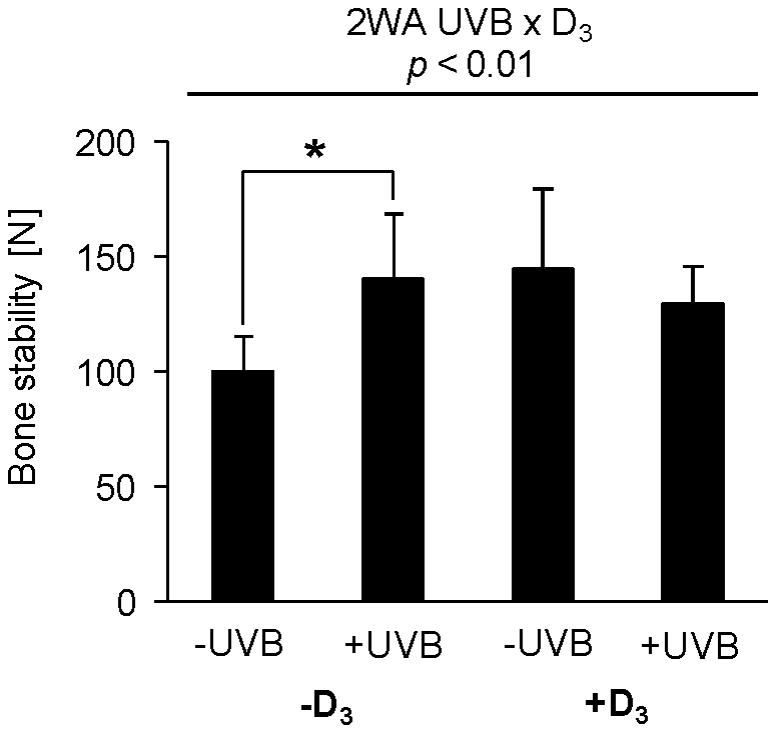
Bone stability in response to UVB exposure and dietary vitamin D_3_. Data in represent mean ± SD (n = 9) of tibiotarsus stability of non-treated (-UVB) or UVB-treated (+UVB) hens that were fed either a vitamin D_3_-deficient (-D_3_) or vitamin D_3_-adequate diet (+D_3_), respectively. Data were analyzed by two-way ANOVA. Classification factors were UVB exposure, vitamin D_3_ in the diet, and the interaction between both factors. Effect of vitamin D_3_: *p*<0.10, vitamin D_3_× UVB: *p*<0.01. Individual means between two groups were compared by the unpaired Student’s t-test, **p<0.01.

Hens non-exposed to UVB radiation that received the vitamin D_3_-deficient diet revealed a lower mechanical stability of tibiotarsus than hens from the other groups (Figure 6, *p*<0.01). Two-way ANOVA data revealed an interactive effect of dietary vitamin D_3_ and UVB exposure on tibiotarsus stability (*p*<0.01, Table S1). In order to investigate possible effects of UVB radiation on folate status, the concentrations of folate in plasma and liver of the hens were determined. Neither the concentration of folate in plasma (-UVB/−D_3_ group, 49.8±18.6 nmol/l; -UVB/+D_3_ group, 45.5±17.4 nmol/l; +UVB/−D_3_ group, 56.8±21.5 nmol/l; +UVB/+D_3_ group, 41.5±10.4 nmol/l; mean ± SD), nor that in liver (-UVB/−D_3_ group, 10.5±1.1 µg/g; -UVB/+D_3_ group, 9.8±2.3 µg/g; +UVB/−D_3_ group, 10.1±1.7 µg/g; +UVB/+D_3_ group, 10.5±1.4 µg/g; mean ± SD) was influenced by dietary vitamin D_3_ and UVB exposure, respectively. This was confirmed by the two-way ANOVA data (Table S1).

## Discussion

Results from the current study reveal UVB exposure of hens as an appropriate way and a highly effective approach to increase the vitamin D content mainly in eggs and also in meat. Data further show that an exposure to UVB is capable of raising the vitamin D content in egg yolk and muscle much stronger than feeding hens with diets that contain maximum permissible dosages of vitamin D_3_. UVB radiation was still effective in increasing the vitamin D content of eggs and meat even in the group that received 3,000 IU vitamin D_3_/kg feed. Previous studies that aimed to increase the vitamin D_3_ content in eggs used vitamin D_3_-enriched feeds. In these experiments the transfer of vitamin D_3_ from the feed into eggs proved to be very efficient and highly responsive [24], [25], [26]. In one of these studies in which hens received diets with 9,700, 17,200, 24,700 and 102,200 IU vitamin D_3_ per kg diet, the peak vitamin D_3_ contents of egg yolk were 22, 41, 60 and 870 µg/100 g (wet basis), respectively [24]. In Europe, the maximum amount of supplemented vitamin D_3_ specified by the Council of the European Communities (Council Directive 70/524/EEC) is set to a quantitative limit of 3,000 IU per kg feed for laying hens. This means that beyond this limit further diet-induced increases of vitamin D_3_ content in eggs are not feasible. Our findings show that exposure of chickens to UVB radiation or natural sunlight seems to offer a promising alternative to fortify foods with vitamin D. Assuming that an average-sized egg comprised of 7 g yolk dry matter, an egg from an UVB exposed hen on a vitamin D_3_-adequate diet would provide on average 2.5 µg vitamin D (vitamin D_3_+25(OH)D_3_) compared to eggs from non-exposed hens on the same diet which contained 0.55 µg. Vitamin D_3_ analysis reveal that meat from the +D_3_/+UVB group contained 0.7 µg vitamin D/100 g compared to meat of the +D_3_/−UVB group that contained 0.2 µg/100 g.

In 2008, Ko et al. already established UVB radiation as a method to increase the vitamin D_2_ content in sliced shiitake and white button mushrooms [27]. In that study, an UVB radiation dose of 75 kJ/m^2^ increased the vitamin D_2_ contents in gill of shiitake mushrooms from less than 500 µg/100 g in the non-exposed mushrooms up to 6,000 µg/100 g in the radiated mushrooms. Sliced button mushrooms exposed to UVB doses of 30 kJ/m^2^ revealed a vitamin D_2_ content of 3,500 µg/100 g. Although irradiation of mushrooms seems to be highly efficient in vitamin D_2_ fortification, it should be taken into consideration that the final vitamin D contents per gram food were extremely high and probably of hazardous nature. In contrast to mushrooms, the efficiency of vitamin D_3_ synthesis in the skin of animals is not only influenced by UV intensity but also by skin pigmentation and the thickness of hair coat, feathers or horny scales. Currently, there are only few published data that investigated the effectiveness of solar and UV irradiation in raising the levels of vitamin D_3_ in animals and animal products (see review [28]). For example, Ferguson et al. have shown that vitamin D content of eggs from chameleons increased in a dose-dependent manner in response to UVB exposure, and Kurmann & Indyk demonstrated lower vitamin D concentrations in bovine milk in winter (2.4 µg per g milk fat) than in summer (9.2 µg per g milk fat) [29], [30]. We assume that the body part which is exposed to UVB is an essential factor for the efficacy of vitamin D fortification. Data of the current study reveal strong variations of 7-DHC concentrations in the different skin areas of hens, with remarkably high 7-DHC levels in the unfeathered skin of legs that were on average 190 times higher than that of comb skin. The important role of the chicken legs for synthesis of vitamin D_3_ has been already reported by Tian and co-workers who found 30 times higher 7-DHC concentrations in skin of the legs and feet of chickens than in body skin [31]. Thus, we assume that “bio-addition” of vitamin D in eggs via UVB radiation only works if the location of the UVB lamps guarantees an irradiation of the feet skin.

An interesting finding of this study was that UVB exposure could increase vitamin D_3_ and 25(OH)D_3_ concentrations in egg yolk and muscle, whilst an oral administration of vitamin D_3_ mainly increased 25(OH)D_3_, but had minor impact on vitamin D_3_ in egg yolk and muscle. 25(OH)D_3_ is primarily synthesized in liver by 25-hydroxylation of vitamin D_3_ from endogenous synthesis or diet. The hepatic 25-hydroxylation is not strictly feedback regulated and therefore mainly reflects vitamin D_3_ status [32]. This relationship was confirmed by the observation that the concentrations of 25(OH)D_3_ in plasma, egg yolk and muscle increased significantly with dietary vitamin D_3_ and also UVB exposure. Domestic fowls synthesize two vitamin D_3_-binding proteins, one that binds 25(OH)D_3_, and the other which mainly binds vitamin D_3_ [19]. It is suggested that the selective mechanism that incorporates vitamin D_3_ into yolk gives the chick embryo the opportunity to control its own 25(OH)D_3_ supply [33]. UVB irradiation of farm animals seems to provide a safe approach to increase vitamin D_3_ without running the risk of vitamin D_3_ overdose. In the case of intense UV irradiation or if animals are exposed to excessive or prolonged exposure to sun, previtamin D_3_ and vitamin D_3_ photoisomerizes to biologically inactive tachysterol and lumisterol, which are desquamated with keratinocytes during normal skin turnover [6], [33], [34]. Health and performance data further indicate no symptoms of erythema, behavioral disorders or an impaired folate status in consequence of the applied UV treatment. The folate concentrations in plasma and liver of the hens were analyzed since UV irradiation is known to be capable of degrading folate in human blood and skin [35], [36]. UVA radiation (315–400 nm) is suggested to be mainly responsible for this effect because it has a greater dermal penetration depth, and can degrade the biological form of folate, 5-methyltetrahydrofolate (5MTHF), in dermal circulation by generation of reactive oxygen species [37], [38], [39], [40]. Other mechanisms such as the direct degradation of folate in the blood by UVA may also contribute to impact folate status [35], [37]. In contrast, UVB radiation (280–315 nm) is unable to penetrate into the dermal circulation and has therefore presumably a lower potential to impact blood levels of folate [35], [38], [39]. Plasma and liver folate data of the current experiment confirm no adverse effect of UVB radiation on folate status, although it should be considered that the period of UVB exposure was relatively short.

This study further reveals that UVB irradiation is capable of optimizing laying performance, egg shell quality, and bone stability in hens that received no vitamin D_3_ with their diet. Although hens from the +D_3_/+UVB group had significantly higher plasma levels of 25(OH)D_3_ and 1,25(OH)_2_D_3_ then hens from the +D_3_/−UVB group, laying performance, egg weight, egg shell thickness, and egg shell stability could not be further improved by the additional treatment with UVB radiation. This is in accordance with previous data that did not show any additional effect of UV radiation on laying performance and egg shell quality in breeders supplemented with sufficient amount of vitamin D_3_ [41].

### Conclusions

In conclusion, the current study shows that UVB exposure of chickens that ensures irradiation of the whole body, including legs, is highly effective in increasing the vitamin D concentration in eggs, and also meat. We therefore consider UVB treatment of farmed animals as an effective and novel approach for “bio-addition” of foods with vitamin D. Considering the option that free-ranged chickens are still exposed to natural sun light, free-range husbandry could become a cheap alternative to the artificial UVB irradiation to produce vitamin D_3_ fortified eggs.

## Supporting Information

Table S1 Two-way analysis of variance table for the chicken and egg data.
(DOCX)
