# Supplementary material for: UVB Exposure of Farm Animals: Study on a Food-Based Strategy to Bridge the Gap between Current Vitamin D Intakes and Dietary Targets
Source: PLoS One. 2013 Jul 24;8(7):e69418. doi: 10.1371/journal.pone.0069418 (PMC3722170; doi:10.1371/journal.pone.0069418)
Supplement: Table S1 — Two-way analysis of variance table for the chicken and egg data. (DOCX) [file pone.0069418.s001.docx]

**Table S1**  Two-way analysis of variance table for the chicken and egg data.

|  |  | **Two-way ANOVA (*p* value)** | | |
| --- | --- | --- | --- | --- |
|  |  | UVB | D_3_ | UVB x D_3_ |
| **Food intake** | week 1 | 0.222 | 0.630 | 0.996 |
|  | week 2 | 0.525 | 0.439 | 0.361 |
|  | week 3 | 0.822 | 0.851 | 0.078 |
|  | final | 0.256 | 0.345 | 0.067 |
| **Body weight** | basal | 0.078 | 0.219 | 0.117 |
|  | week 1 | 0.079 | 0.118 | 0.145 |
|  | week 2 | 0.072 | 0.121 | 0.162 |
|  | week 3 | 0.074 | 0.421 | 0.048 |
|  | final | 0.051 | 0.518 | 0.063 |
| **Plasma 25(OH)D_3_** | basal | 0.682 | 0.134 | 0.642 |
|  | final | 0.000 | 0.000 | 0.000 |
| **Plasma 1,25(OH)_2_D_3_** | basal | 0.513 | 0.120 | 0.912 |
|  | final | 0.009 | 0.000 | 0.390 |
| **Plasma calcium** | basal | 0.714 | 0.481 | 0.175 |
|  | final | 0.482 | 0.089 | 0.607 |
| **Plasma inorganic phosphate** | basal | 0.993 | 0.339 | 0.608 |
|  | final | 0.592 | 0.111 | 0.810 |
| **Vitamin D_3_ in egg yolk** | basal | 0.937 | 0.622 | 0.212 |
|  | final | 0.000 | 0.000 | 0.036 |
| **25(OH)D_3_ in egg yolk** | basal | 0.760 | 0.906 | 0.181 |
|  | final | 0.000 | 0.000 | 0.000 |
| **Vitamin D_3_ in muscle** | final | # | # | # |
| **25(OH)D_3_ in muscle** | final | # | # | # |
| **Egg weight** | basal | 0.672 | 0.225 | 0.825 |
|  | week 1 | 0.686 | 0.873 | 0.361 |
|  | week 2 | 0.728 | 0.991 | 0.587 |
|  | week 3 | 0.918 | 0.807 | 0.220 |
|  | final | 0.395 | 0.730 | 0.385 |
| **Egg shell thickness** | basal | 0.792 | 0.220 | 0.541 |
|  | week 1 | 0.228 | 0.660 | 0.306 |
|  | week 2 | 0.216 | 0.035 | 0.019 |
|  | week 3 | 0.226 | 0.008 | 0.064 |
|  | final | 0.121 | 0.004 | 0.053 |
| **Egg shell stability** | basal | 0.498 | 0.468 | 0.993 |
|  | week 1 | 0.608 | 0.424 | 0.872 |
|  | week 2 | 0.018 | 0.224 | 0.872 |
|  | week 3 | 0.070 | 0.091 | 0.171 |
|  | final | 0.005 | 0.018 | 0.046 |
| **Bone stability** | final | 0.146 | 0.062 | 0.003 |
| **Plasma folate** | basal | 0.232 | 0.402 | 0.371 |
|  | final | 0.800 | 0.114 | 0.366 |
| **Liver folate** | final | 0.997 | 0.663 | 0.441 |

^#^Two-way ANOVA analysis not possible because of non-detectable vitamin D_3_ and 25(OH)D_3_ in muscles of the –UVB/-D_3_ and –UVB/+D_3_ group, respectively. Detection limit was 0.17 µg/100 g for vitamin D_3_, and 0.1 µg/100 g for 25(OH)D_3_.
